# Polycomb repressor complex 2 regulates HOXA9 and HOXA10, activating ID2 in NK/T-cell lines

**DOI:** 10.1186/1476-4598-9-151

**Published:** 2010-06-17

**Authors:** Stefan Nagel, Letizia Venturini, Victor E Marquez, Corinna Meyer, Maren Kaufmann, Michaela Scherr, Roderick AF MacLeod, Hans G Drexler

**Affiliations:** 1 Dept. of Human and Animal Cell Lines, DSMZ - German Collection of Microorganisms and Cell Cultures, Inhoffenstr. 7B, 38124 Braunschweig, Germany; 2 Dept. of Hematology, Hemostasis, Oncology and Stem Cell Transplantation, Medical School Hannover, Carl-Neubergstr. 1, 30125 Hannover, Germany; 3 National Institutes of Health, National Cancer Institute, Laboratory of Medicinal Chemistry, Frederick, Maryland, USA

## Abstract

**Background:**

NK- and T-cells are closely related lymphocytes, originating from the same early progenitor cells during hematopoiesis. In these differentiation processes deregulation of developmental genes may contribute to leukemogenesis. Here, we compared expression profiles of NK- and T-cell lines for identification of aberrantly expressed genes in T-cell acute lymphoblastic leukemia (T-ALL) which physiologically regulate the differentiation program of the NK-cell lineage.

**Results:**

This analysis showed high expression levels of HOXA9, HOXA10 and ID2 in NK-cell lines in addition to T-cell line LOUCY, suggesting leukemic deregulation therein. Overexpression experiments, chromatin immuno-precipitation and promoter analysis demonstrated that HOXA9 and HOXA10 directly activated expression of ID2. Concomitantly elevated expression levels of HOXA9 and HOXA10 together with ID2 in cell lines containing MLL translocations confirmed this form of regulation in both ALL and acute myeloid leukemia. Overexpression of HOXA9, HOXA10 or ID2 resulted in repressed expression of apoptosis factor BIM. Furthermore, profiling data of genes coding for chromatin regulators of homeobox genes, including components of polycomb repressor complex 2 (PRC2), indicated lacking expression of EZH2 in LOUCY and exclusive expression of HOP in NK-cell lines. Subsequent treatment of T-cell lines JURKAT and LOUCY with DZNep, an inhibitor of EZH2/PRC2, resulted in elevated and unchanged HOXA9/10 expression levels, respectively. Moreover, siRNA-mediated knockdown of EZH2 in JURKAT enhanced HOXA10 expression, confirming HOXA10-repression by EZH2. Additionally, profiling data and overexpression analysis indicated that reduced expression of E2F cofactor TFDP1 contributed to the lack of EZH2 in LOUCY. Forced expression of HOP in JURKAT cells resulted in reduced HOXA10 and ID2 expression levels, suggesting enhancement of PRC2 repression.

**Conclusions:**

Our results show that major differentiation factors of the NK-cell lineage, including HOXA9, HOXA10 and ID2, were (de)regulated via PRC2 which therefore contributes to T-cell leukemogenesis.

## Introduction

Adult lymphopoiesis starts with progenitor cells which originate from CD34+ hematopoietic stem cells (HSC) in the bone marrow. While the development of natural killer (NK)- cells completes primarily in the bone marrow, T-cells finalize their differentiation in the thymus [1-3]. Nevertheless, the facts that NK-cell differentiation also occurs in the thymus and early thymocytes exhibit the capacity to differentiate into NK-cells demonstrate a close developmental relationship between these two lymphocytic lineages [4]. Early steps in lymphocytic differentiation are principally (but not exclusively) regulated by members of the basic helix-loop-helix (bHLH) family of transcription factors, including TCF3/E2A and TCF12/HEB. Downregulation of their activity by oncogenic family members TAL1 or LYL1 contributes to T-cell leukemogenesis [5-7]. Physiological expression of inhibitory bHLH protein ID2 regulates early developmental processes of NK-cells while ectopic expression of ID2 inhibits those in T-cells [8-10]. Another group of T-cell acute lymphoblastic leukemia (T-ALL)-associated oncogenes are homeobox genes and includes members of the NK-like family, TLX1/HOX11, TLX3/HOX11L2 and NKX2-5/CSX [11-13], and of the clustered homeobox genes, HOXA5, HOXA9, HOXA10 and HOXA11 [14,15].

Chromosomal juxtaposition of the HOXA gene cluster with T-cell receptor (TCR)-beta via inv(7)(p15q34) or t(7;7)(p15;q34) results in ectopic expression of several HOXA genes [14,15]. Translocations fusing the mixed lineage leukemia (MLL) locus with diverse partner genes also mediate HOXA gene deregulation in both, acute myeloid leukemia (AML) and ALL [16-18]. MLL is a chromatin activator which embodies histone-methyltransferase (HMT) activity affecting histone H3 at position K4 [19]. Vertebrates possess 4 MLL homologues which share sequence similarity and this specific HMT activity with the related SET1 proteins [20]. Moreover, the fusion protein SET-NUP214 which originates by the cryptic chromosomal aberration del(9)(q34q34) in T-ALL mediates HOXA activation by H3 methylation at position K79 via recruitment of HMT DOTL1 [21]. Thus, deregulation of HOXA genes in T-ALL may be performed either directly by chromosomal rearrangements or indirectly by the aberrant activities of chromatin activators.

These activators compete with repressor complexes, consisting of polycomb group proteins. Two distinct polycomb repressor complexes (PRC), PRC1 and PRC2, have been identified, comprising, firstly, BMI1 together with CBX4 and, secondly, EED together with EPC1, EZH2 and SUZ12 [22-24]. EZH2 is another type of HMT which methylates histone H3K27 to mediate gene repression [25,26]. Thus, two functional types of chromatin complexes, activators and repressors, regulate the expression of HOXA genes by differing methylation of histone H3.

The aim of our study was to identify developmental oncogenes and their deregulating mechanisms in T-ALL cells. Therefore, we compared gene expression profiles of NK- and T-cell lines and identified the conspicuous expression of HOXA9, HOXA10 and ID2 which may represent the physiological situation in the differentiation process of NK-cells but aberrant activity in one T-ALL cell line. Analysis of genes, coding for chromatin activators/repressors, revealed the (de)regulatory impact of two PRC2 components in lymphoid HOXA gene expression.

## Materials and methods

### Cells and treatments

Cell lines were supplied by the DSMZ (Braunschweig, Germany). Cultivation was performed as described by Drexler [27]. For stimulation of cell lines we used 3-Deazaneplanocin A (DZNep) which was synthesized at the National Institutes of Health, and 5-Aza-2'-deoxycytidine (AZA) and rapamycin which were obtained from Sigma (Taufkirchen, Germany). Primary CD34+ cells were obtained from peripheral blood of a healthy donor and isolated using the MACS system for cell preparations according to the manufacturers' protocol (Miltenyi Biotec, Bergisch Gladbach, Germany).

### RNA extraction and cDNA synthesis

Total RNA was extracted from cells using TRIzol reagent (Invitrogen, Karlsruhe, Germany). cDNA was subsequently synthesized from 5 μg RNA by random priming, using Superscript II (Invitrogen).

### Expression profiling

For quantification of gene expression via profiling we used DNA chips U133A Plus 2.0 obtained from Affimetrix, (Buckinghamshire, UK). Chip-data analysis was performed as described recently [28]. Analysis of expression data was performed using online programs. For creation of heat maps and similarity trees we used CLUSTER version 2.11 and TREEVIEW version 1.60 http://rana.lbl.gov/EisenSoftware.htm.

### Polymerase chain reaction (PCR) analysis

Quantitative expression analysis was performed using the 7500 Real-time System, commercial buffer and primer sets for BIM, EZH2, HOP, HOXA9, HOXA10, KCNQ1 and RUNX2 (Applied Biosystems, Darmstadt, Germany). Expression analysis of pri-miR-17-92 was performed as described recently [29]. For normalization of expression levels we used TBP. Quantitative analyses were performed in triplicate and repeated twice.

The following oligonucleotides were used for reverse transcription (RT)-PCR were generated by MWG Eurofins (Ebersberg, Germany): miR26-for: 5'-CCGGTTGAAATCGATGGAAC-3'; miR26-rev: 5'-TCAGGTCCTTCACGTAGTTC-3'; miR101-for: 5'-TCCTCATGCAAATAGCGGGAAG-3'; miR101-rev: 5'-AAGCCATGGCATTGCAGTCCTC-3'; TEL-for: 5'-AGGCCAATTGACAGCAACAC-3'; TEL-rev: 5'-TGCACATTATCCACGGATGG-3'; TFDP1-for: 5'-TCTTCTCTGGGAAGGTGAAC-3'; TFDP1-rev: 5'-TCCTACCACCAGGGTGTTTG-3'. Obtained PCR products were analyzed by agarose gel electrophoresis.

### ID2 promoter analysis

To quantify the impact of HOXA10 on the ID2 promoter we cloned a corresponding genomic fragment containing the HOXA10 binding site in front of a reporter gene. PCR using oligonucleotides ID2-for (5'-GCAAGCTTATCTAGCCCTCCCTCTAGCTG-3') and ID2-rev (5'-AAGGATCCTGACAGATAGGTGGCCCTAGC-3') generated a promoter fragment of 1550 bp in length. The validity of the construct was confirmed by sequence analysis (MWG Eurofins). As described previously, the reporter gene consists of a genomic fragment of the HOXA9 gene, comprising exon1-intron-exon2, allowing the quantification of the transcribed and spliced reporter mRNA via RQ-PCR [30]. The reporter gene was cloned via *EcoRI*, the promoter via *HindIII/BamHI *into the backbone of the pcDNA3 vector [30]. A cotransfected luciferase construct served as transfection control, quantified by the Luciferase Assay System (Promega, Mannheim, Germany) using the luminometer Lumat LB9501 (Berthold Technologies, Bad Wildbad, Germany).

### Fluorescence In Situ Hybridization (FISH)

FISH analysis was performed as described recently [31]. The following RP11-clones were obtained from the (now defunct) clone facility at the Sanger Centre, (Cambridge, UK): 892G20 and 355H10 for ID2; 627P22 and 812K17 for HOXA; 375K6 and 11H19 for RUNX2. Fluorescence images were captured using an Axioskop 2 plus microscope (Zeiss, Göttingen, Germany) and analyzed with Cytovision 2 software (Applied Imaging, Newcastle, UK).

### Gene transfer

Expression constructs for HOXA9, HOXA10, ID2 and TFDP1 (in pCMV-XL5/4 vectors) were obtained from Origene (Wiesbaden, Germany). Transfection of expression constructs into cervix carcinoma cell line HELA was performed using transfection reagent SuperFect (Qiagen, Hilden, Germany). Transfection experiments were performed at an efficiency of about 30% as determined by fluorescence microscopy. Expression construct for ID2 and siRNAs directed against EZH2 and HOP (Qiagen) were electroporated into cell lines using the EPI-2500 impulse generator (Fischer, Heidelberg, Germany). Transfection and electroporation experiments have been performed twice.

VSV.G-pseudotyped lentiviral particles were generated by calcium phosphate co-transfection of embryonal kidney 293T cells and viral supernatants were concentrated as previously described [32]. Lentiviral transduction of T-ALL cell line JURKAT was performed with a multiplicity of infection of approximately two. JURKAT-HOXA9 cells showed low transduction efficiency and were subsequently sorted, using a FACSAria cell sorter (BD Bioscience, Heidelberg, Germany). The transduction efficiency of LOUCY-TFDP1 was about 60%.

### Chromatin immuno-precipitation (ChIP)

ChIP analysis was performed with the ChIP Assay Kit (Millipore-Upstate, Schwalbach, Germany). Antibody anti-HOXA10 was obtained from Santa Cruz Biotechnology (Heidelberg, Germany). The subsequent PCR analysis was carried out, using nested ID2-oligonucleotides.

For the HOXA10-binding site: ID2-for: 5'-TATTGGGCGTGCTGAAACAG-3', ID2-rev: 5'-GATTAAGGCAGTGCCTTCTC-3', ID2-for-nested: 5'-CACTAGTAACTTAGGCCTCG-3', ID2-rev-nested: 5'-CCCTGATGTTAGTAAAATGGC-3'.

For control: ID2-for: 5'-CCAGGGTGTTCTCTTACTTG-3', ID2-for-nested: 5'-GCCCTTTCTGCAGTTGGA-3', ID2-rev: 5'-CAGCATTCAGTAGGCTTGTG-3', ID2-rev-nested: 5'-GGATCCTTCTGGTATTCA-3'.

### Protein analysis

For protein analysis Western blotting was performed as described previously [13]. Briefly, proteins obtained from cell lysates were transferred semi-dry onto nitrocellulose membranes (Bio-Rad, München, Germany) which were blocked with 5% bovine serum albumin (BSA) dissolved in phosphate-buffered-saline (PBS) buffer. Antibodies to the following proteins were obtained from Santa Cruz Biotechnology: E2F1, ERK1/2, ID2, STAT5; and from Cell Signaling (Freiburg, Germany): EZH2.

### MTT assay

Cell lines were treated for 16 h with 100 μM etoposide (Sigma), which has been dissolved in dimethylsulfoxide, and subsequently prepared for standardized MTT (3-(4,5-dimethylthiazol-2-yl)-2,5-diphenyltetrazolium bromide; obtained from Sigma) assays. The absorbance was determined at 570 nm by an ELISA reader (Thermo Electron, Vantaa, Finland).

## Results

### Expression analysis of NK- and T-cell lines

**Figure 1:**
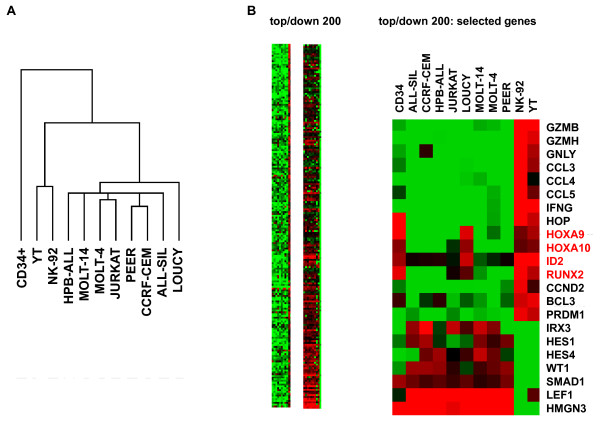
**Gene expression analysis by profiling**. (A) Expression profiling data were analyzed by cluster analysis. Of note, all cells were ordered according to their origin. LOUCY was the most varied T-cell line. (B) Expression profiling data were transformed into a heat map, demonstrating 200 top/down expressed genes in NK/T-cell lines and CD34+ HSCs. Red indicates high, green low and black medium expression levels. Selected top/down expressed genes are shown on the right, including HOXA9, HOXA10, ID2 and RUNX2 highlighted in red.

Two NK-cell lines, NK-92 and YT, together with eight T-cell lines and one sample of primary CD34+ HSC were examined by expression profiling and subsequently by cluster analysis (Fig. 1A). Three main clusters were discerned, comprising HSC, NK-cell lines and T-cell lines, respectively, representing progenitor cells and two lymphocytic differentiation lineages. Of note, LOUCY was the most divergent T-cell line, indicating substantial differences in gene activities.

**Figure 2:**
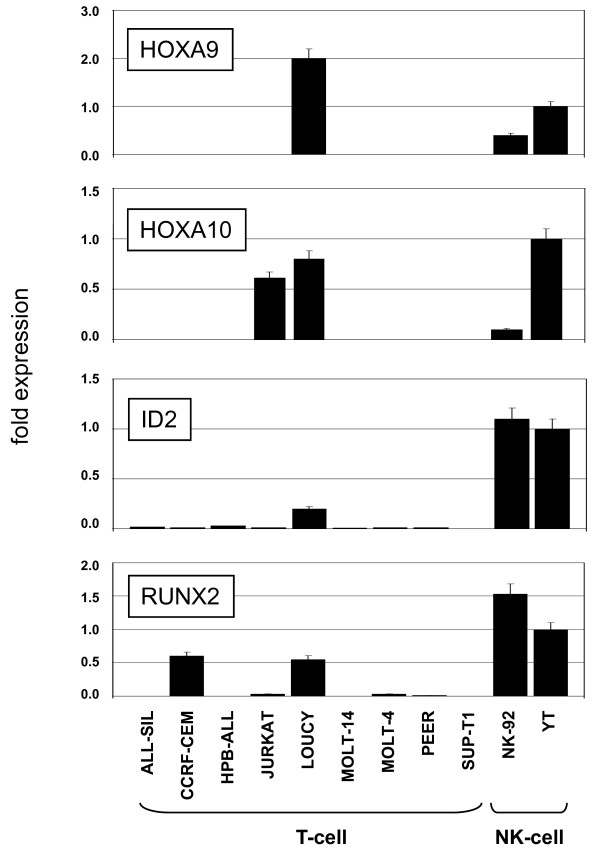
**Gene expression analysis by real-time PCR**. Quantitative RT-PCR analysis of candidate genes HOXA9, HOXA10, ID2 and RUNX2 in selected T- and NK-cell lines. Of note, high expression levels of all four genes were confirmed in NK-92, YT and LOUCY. Expression levels of YT were set to 1.

To identify specifically expressed genes within the NK-cell lineage we subtracted averaged values of NK- and T-cell data sets, respectively, and ranked these differences to generate a list of differentially expressed genes. From this list we analyzed the top-200 over- and under-expressed genes as heat maps (Fig. 1B). These candidates included genes involved in immunological defence such as granzymes (GZMB, GZMH) and interferon gamma (IFNG) in addition to several differentiation factors (HOXA9, HOXA10, ID2, RUNX2). Interestingly, LOUCY resembled NK-cell lines by displaying high expression levels of HOXA9, HOXA10, ID2 and RUNX2, suggesting deregulation of these NK-lineage genes within this T-ALL cell line [8,9,33-36]. Quantitative RT-PCR (RQ-PCR) analysis of HOXA9, HOXA10, ID2 and RUNX2 confirmed their expression in NK-cell lines and LOUCY (Fig. 2). Additionally, JURKAT cells also expressed HOXA10 and CCRF-CEM expressed RUNX2. Taken together, we identified in T-ALL cell line LOUCY coexpression of four developmental genes which are involved in NK-cell lineage differentiation.

### HOXA9 and HOXA10 activate expression of ID2

We subsequently focused on potential mechanisms which might conceivably deregulate these candidate oncogenes in LOUCY. FISH analysis using flanking probes excluded chromosomal translocations at 7p15, 2p25 and 6p12 which might have been responsible for high expression levels of HOXA9/HOXA10, ID2 and RUNX2, respectively (data not shown). Sequence analyses of promoter regions, according to genome browser data of the University of California Santa Cruz (UCSC), demonstrated binding sites for HOXA9/HOXA10 within the promoter regions of ID2 (at -2167 bp) and of RUNX2 (at -5249 bp) which may indicate regulative interactions. Accordingly, in osteoblasts HOXA10 has been shown to activate expression of RUNX2 [37]. Therefore, we decided to investigate potential oncogenic deregulations of ID2 and/or RUNX2 by HOXA9 and HOXA10 in T-ALL cells.

**Figure 3:**
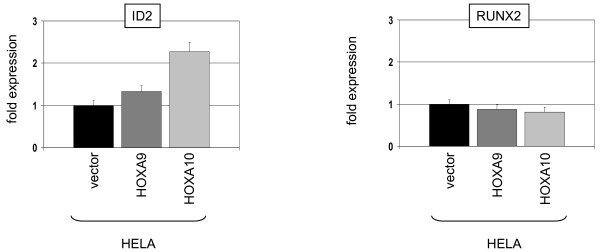
**Expression analysis in HELA cells**. HELA cells were transfected with expression constructs for HOXA9 and HOXA10 in addition to a vector control. Subsequent RQ-PCR analysis of ID2 and RUNX2 demonstrated elevated ID2 transcript levels while that of RUNX2 remained unchanged.

First, we transfected HELA cells with CMV-driven constructs for overexpression of transcription factors HOXA9 or HOXA10. Subsequent RQ-PCR analysis demonstrated elevated expression levels of ID2 in HOXA9 and HOXA10 transfected cells. However, the expression levels of RUNX2 showed no significant alteration, failing to support a regulatory connection (Fig. 3). Thus, these data showed that in contrast to RUNX2, both HOXA9 and HOXA10 activated expression of ID2 in HELA.

**Figure 4:**
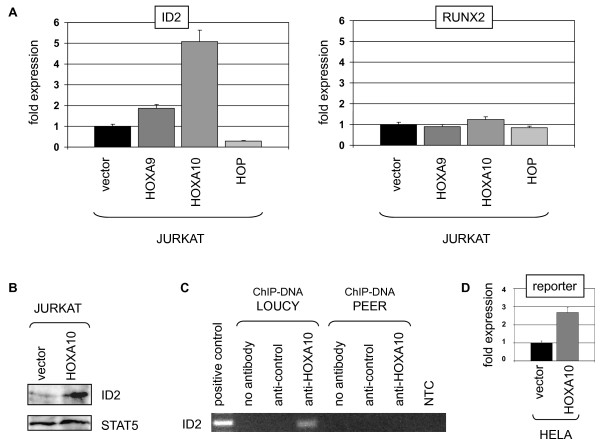
**Expression analysis in T-ALL cells**. (A) JURKAT cells were lentivirally transduced for overexpression of HOXA9, HOXA10 and HOP, respectively. Subsequent RQ-PCR analysis of ID2 and RUNX2 demonstrated elevated ID2 transcription in HOXA9/HOXA10 overexpressing cells and reduced levels in HOP overexpressing cells. Expression levels of RUNX2 showed no alterations. (B) Western blot analysis demonstrated elevated expression of ID2 protein in JURKAT cells overexpressing HOXA10. STAT5 protein analysis served as loading control. (C) ChIP-PCR analysis in LOUCY (HOXA10-positive) and PEER (HOXA10-negative), using HOXA10 antibody demonstrated direct binding of HOXA10 to the ID2 promoter region at -2167 bp. (D) HELA cells were cotransfected with an ID2 promoter-construct and an expression-construct for HOXA10 or vector control. Reportergene activity was subsequently quantified by real-time PCR, indicating an activatory input by HOXA10.

To analyze this mechanism in T-ALL we lentivirally transduced JURKAT cells with constructs of HOXA9 or HOXA10 for overexpression. Subsequent expression analysis by RQ-PCR demonstrated elevated and unaltered levels of ID2 and RUNX2, respectively (Fig. 4A). Greater levels of ID2 activation were effected by HOXA10 than by HOXA9, correlating with the results obtained in HELA. Western blot analysis confirmed elevated ID2 expression at the protein level in HOXA10 overexpressing cells (Fig. 4B). Moreover, ChIP analysis of the ID2 promoter region at -2167 bp in LOUCY cells demonstrated binding of HOXA10 protein (Fig. 4C), indicating a direct activation mechanism by these homeodomain proteins in T-ALL cells. Finally, we used a reporter gene construct combined with a corresponding fragment of the ID2 promoter, containing the indicated HOXA10 binding site. Cotransfection together with HOXA10 expression construct into HELA cells resulted in enhanced reporter gene activity, as compared to the vector control and analyzed by RQ-PCR. These results support the direct input of HOXA10 in ID2 expression (Fig. 4D).

**Figure 5:**
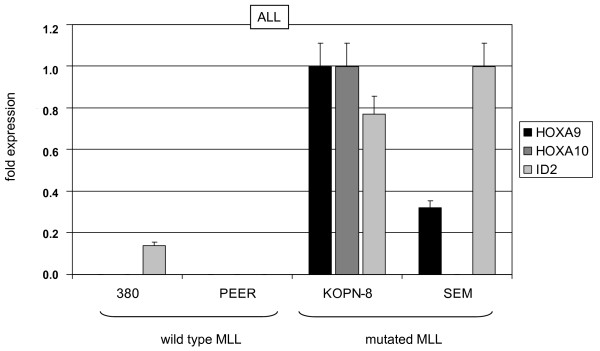
**Expression analysis in ALL cell lines**. RQ-PCR analysis of HOXA9, HOXA10 and ID2 demonstrated higher expression levels in ALL cell lines carrying MLL-translocations. The highest expression levels of each gene were set to 1.

**Figure 6:**
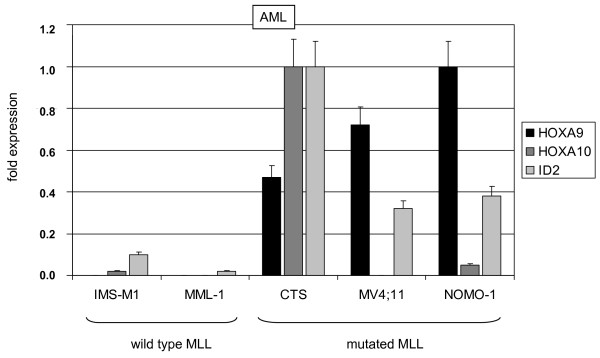
**Expression analysis in AML cell lines**. RQ-PCR analysis of HOXA9, HOXA10 and ID2 demonstrated higher expression levels in ALL cell lines carrying MLL-translocations. The highest expression levels of each gene were set to 1.

Both, HOXA9 and HOXA10 are targets of MLL-fusion proteins in AML and ALL malignancies [16]. RQ-PCR analysis of HOXA9, HOXA10 and ID2 in MLL-translocation-positive and -negative cell lines of AML or ALL origin [18], respectively, indicated a positive correlation between expression of HOXA9/HOXA10 and ID2 (Fig. 5, 6). These results confirmed the activating impact of HOXA9/HOXA10 on ID2 expression and identified this bHLH gene as indirect downstream target of MLL-fusion proteins.

**Figure 7:**
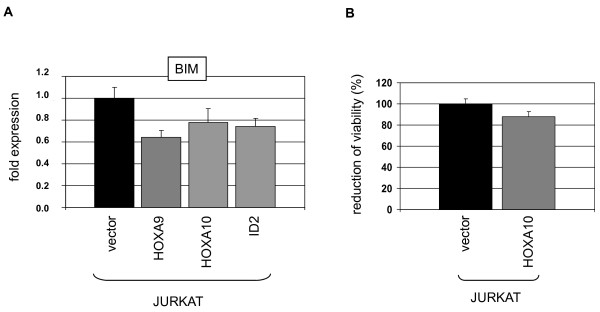
**Target gene analysis of ID2**. (A) RQ-PCR analysis of BIM demonstrated reduced expression levels in JURKAT cells overexpressing HOXA9, HOXA10 or ID2. (B) MTT assay of transduced JURKAT cells after treatment with etoposide indicated reduced apoptosis by HOXA10 overexpression.

TCF3 controls many cellular processes during thymocyte development, including differentiation and survival. Accordingly, gene expression analysis of TCF3 target genes has identified activation of the pro-apoptosis gene BIM [38]. Since ID2 suppresses the activity of TCF3 [9], we analyzed BIM expression in transduced JURKAT cells. Our data demonstrated downregulation of BIM in HOXA9/HOXA10 and ID2 overexpressing cells (Fig. 7A), suggesting anti-apoptotic effects of these homeodomain proteins mediated via ID2 in T-ALL cells. Accordingly, survival analysis by MTT assay after etoposide treatment indicated a slight reduction of apoptosis by HOXA10 overexpression (Fig. 7B).

### Regulation of HOXA9 and HOXA10 by PRC2

**Figure 8:**
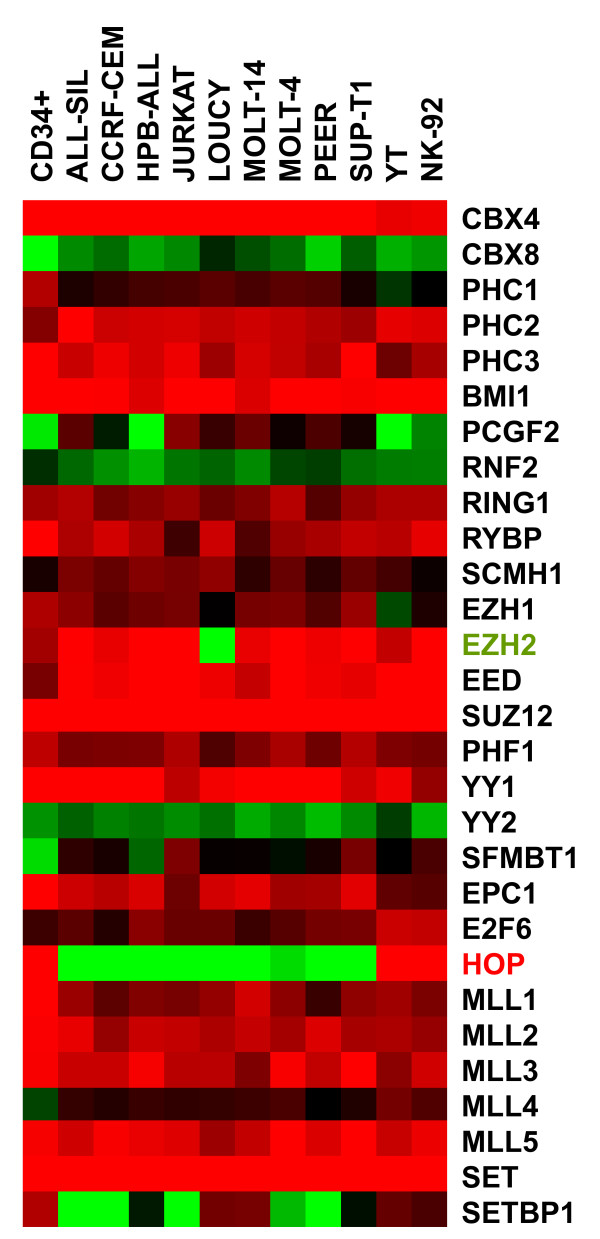
**Gene expression analysis of chromatin genes**. Profiling data of selected chromatin genes were transformed into a heat map, demonstrating reduced expression levels of EZH2 in LOUCY and high expression levels of HOP in NK-cell lines NK-92 and YT. Red indicates high, green low and black medium expression levels.

**Figure 9:**
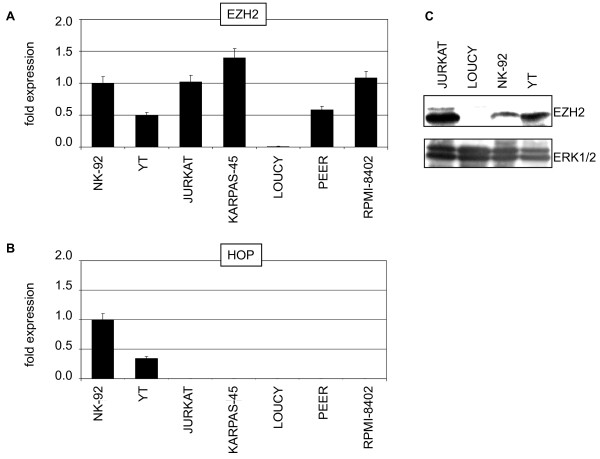
**Expression analysis in NK/T-cells**. (A) RQ-PCR analysis of candidate gene EZH2 in selected T- and NK-cell lines. Of note, reduced EZH2 expression was confirmed in LOUCY. (B) RQ-PCR analysis of candidate gene HOP in selected T- and NK-cell lines. Of note, high expression levels were confirmed in NK-cell lines NK-92 and YT. (C) Western blot analysis of EZH2 protein expression in selected NK/T-cell lines. ERK1/2 protein expression served as loading control. Of note, in LOUCY no EZH2 protein was detectable.

MLL and SET1 proteins together with polycomb-related proteins are chromatin components which regulate transcription of developmental genes, including those of the HOXA cluster [19,20,39-42]. The expression profiling data of their corresponding genes in NK/T-cell lines demonstrated differential transcript levels of PRC2 components EZH2 and homeodomain only protein HOP (Fig. 8). While LOUCY exclusively showed silent EZH2, both NK-cell lines together with HSC expressed HOP in contrast to T-ALL cell lines. The activity of these two genes was subsequently quantified by RQ-PCR in selected cell lines, confirming lack of EZH2 expression in LOUCY and exclusive expression of HOP in NK-cell lines NK-92 and YT (Fig. 9A, B). Additionally, we confirmed absence of EZH2 in LOUCY at the protein level by Western blot analysis (Fig. 9C). These data support the proposed regulatory impact of particular PRC2-proteins in expression of HOXA9 and HOXA10 in NK/T-cells.

**Figure 10:**
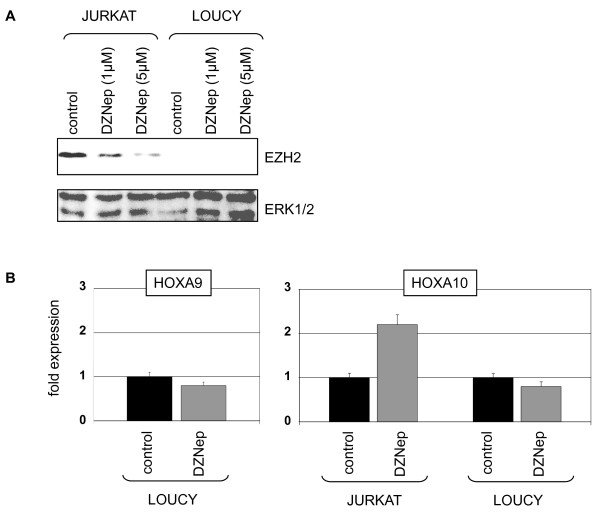
**DZNep treatment of T-ALL cells**. (A) Western blot analysis of EZH2 protein expression in JURKAT and LOUCY cells treated with DZNep. ERK1/2 protein expression served as loading control. Treatment with DZNep reduced concentration dependent the protein levels of EZH2 in JURKAT cells. (B) RQ-PCR analysis of HOXA9 and HOXA10 in LOUCY and JURKAT cells treated with DZNep. Of note, LOUCY showed unaltered expression levels in contrast to JURKAT, demonstrating elevated HOXA10 expression after treatment.

**Figure 11:**
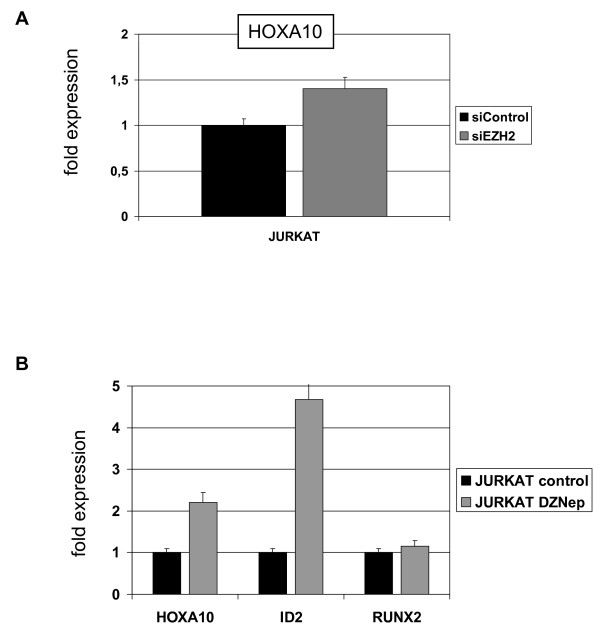
**Suppression of EZH2 in JURKAT**. (A) RQ-PCR analysis of HOXA10 after siRNA-mediated knockdown of EZH2 demonstrated enhanced gene expression. (B) RQ-PCR analysis of HOXA10, ID2 and RUNX2 in JURKAT cells treated with DZNep. Of note, concomitantly with HOXA10, expression levels of ID2 rose after treatment with DZNep in contrast to RUNX2 expression.

Therefore, we treated EZH2-negative LOUCY and EZH2-positive JURKAT cells with DZNep, an inhibitor of EZH2/PRC2 [43]. Western blot analysis confirmed reduction of the EZH2 protein level in treated JURKAT cells (Fig. 10A). Subsequent RQ-PCR analysis of HOXA9 and HOXA10 showed unaltered and increased expression levels, respectively (Fig. 10B). Accordingly, siRNA-mediated knockdown of EZH2 enhanced the expression of HOXA10 in JURKAT (Fig. 11A). Thus, these results confirmed the repressive impact of PRC2-component EZH2 on HOXA-genes in T-cells. Moreover, DZNep treatment resulted in increased expression of HOXA10 downstream target ID2, while that of RUNX2 remained unaltered (Fig. 11B). EZH2/PRC2 is also involved in the genomic imprinting of region 11p15 [44,45]. Here, we analyzed expression of KCNQ1 (located at 11p15) by RQ-PCR after DZNep treatment. While in LOUCY no change of positive KCNQ1 expression level was detected, JURKAT cells demonstrated an induction of KCNQ1 expression, confirming lack of repression by EZH2 in LOUCY and relaxation of repression by DZNep in JURKAT (data not shown).

**Figure 12:**
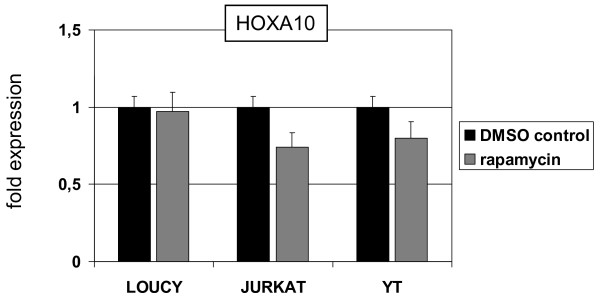
**Expression analysis of HOXA10**. RQ-PCR analysis of HOXA10 in selected NK/T-cell lines after rapamycin treatment. Of note, reduced HOXA10 expression was detected in JURKAT and YT but not in LOUCY.

As shown recently, EZH2 activity is reduced upon phosphorylation via AKT-signaling [46]. To analyze this effect, we treated JURKAT, YT and LOUCY cells with an inhibitor of AKT-signaling, rapamycin, and subsequently quantified HOXA10 expression. Our results demonstrated decreased HOXA10 levels in JURKAT and YT, indicating enhanced EZH2 activity, while those in LOUCY consistently remained unaltered (Fig. 12).

**Figure 13:**
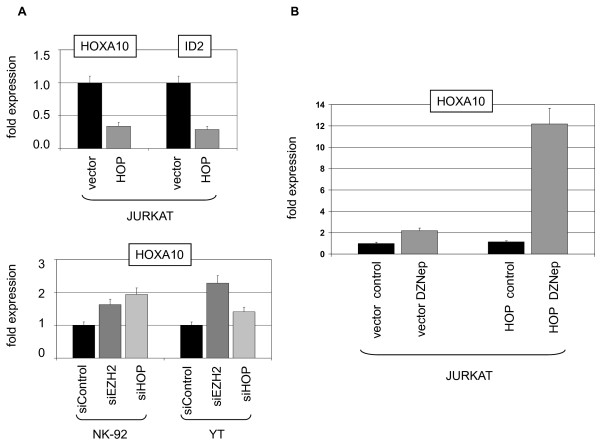
**Effect of HOP in T- and NK-cells**. (A) RQ-PCR analysis in HOP transduced JURKAT cells demonstrated downregulation of HOXA10 and ID2 expression (top). RQ-PCR analysis in siRNA-treated NK-92 and YT cells demonstrated upregulation of HOXA10 expression (below), confirming repressive activity of HOP and EZH2. (B) RQ-PCR analysis in transduced JURKAT cells treated with DZNep, demonstrating enhanced HOXA10 expression in HOP overexpressing cells.

HOP has been shown to interact with PRC2 component EPC1 [47]. To analyze the potential impact on expression of HOXA9/HOXA10 we lentivirally transduced JURKAT cells to overexpress HOP. Subsequent RQ-PCR analysis showed decreased expression levels of HOXA10, indicating the enhancement of PRC2 repression activity. Accordingly, ID2 expression was decreased in HOP-transduced JURKAT cells, while RUNX2 expression remained unchanged (Fig. 13A, Fig. 4A). In NK-92 and YT siRNA-mediated knockdown of HOP or EZH2 resulted in increased expression of HOXA10 (Fig. 13A), confirming their repressive roles in NK-cells as well. Interestingly, DZNep-treatment of HOP-transduced JURKAT cells and corresponding vector-controls demonstrated a much stronger increase of HOXA10 expression in HOP-overexpressing cells (Fig. 13B). This result suggested that HOP enhanced the sensitivity for DZNep, probably by better accessibility.

### Regulation of EZH2 by E2F-factors

To examine possible causes underlying the lack of EZH2 expression in LOUCY cells we looked for chromosomal deletions by consulting genomic array data published by the Sanger centre (Cambridge, UK). However, the EZH2 locus at 7q36 showed no copy number alterations, thus excluding genomic deletion. Moreover, treatment of LOUCY cells with AZA to reactivate DNA-methylated genes showed no effect on EZH2 when analyzed by RQ-PCR (not shown), thus excluding aberrant methylation at this locus. Although, EZH2 is the target of at least two micro-RNAs, namely miR-26 and miR-101 [48,49], RT-PCR analysis showed no differential expression of their corresponding pri-micro-RNAs in LOUCY when compared to control T-ALL cell lines (data not shown).

**Figure 14:**
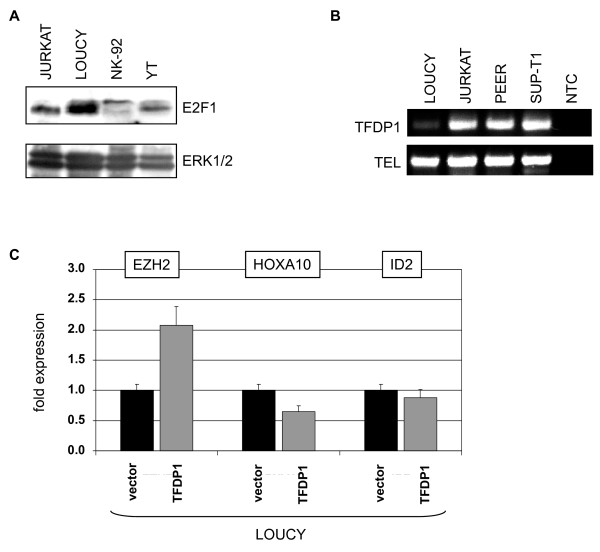
**Regulation of EZH2 expression via E2F/TFDP1**. (A) Western blot analysis of selected NK/T-cell lines demonstrated the presence of E2F1 protein in all cells including LOUCY. (B) RT-PCR analysis of TFDP1 in selected NK/T-cell lines demonstrated reduced expression level in LOUCY. Expression of TEL served as positive control, NTC: no template control. (C) RQ-PCR analysis of LOUCY cells which were lentivirally transduced with an expression construct for TFDP1 demonstrated increased expression levels of EZH2 and decreased levels of HOXA10 and ID2 as compared to vector control cells.

As shown previously, expression of EZH2 is regulated by the E2F1 transcription factor which in turn is deregulated by overexpressed micro-RNAs of the miR-17-92 gene cluster, reducing E2F1 protein levels in T-ALL cells [29,50]. But RQ-PCR analysis failed to indicate enhanced expression level of pri-miR-17-92 (not shown) and western blot analysis detected consistently high amounts of E2F1 protein in LOUCY (Fig. 14A). However, expression profiling analysis of all known E2F-related genes indicated very low levels of TFDP1 in LOUCY which was confirmed by RT-PCR analysis (Fig. 14B). TFDP1 is a cofactor of E2F proteins, acting as heterodimers [51]. Subsequently ectopic expression of TFDP1 in LOUCY cells via lentiviral transduction of a CMV-driven expression-construct resulted in elevated EZH2 expression and accordingly decreased levels of HOXA10 transcripts as analyzed by RQ-PCR. However, the reduction of of ID2 was quite small and not significant (Fig. 13C). In conclusion, these data demonstrate that reduced TFDP1/E2F-activity contributes to the lack of EZH2 expression in this cell line.

## Discussion

T-cell leukemogenesis involves ectopic activation of proto-oncogenes which often regulate physiological development. In this study, comparison of gene expression profiles of NK/T-cell lines identified the apparently aberrant expression of developmental homeobox genes, HOXA9 and HOXA10, a bHLH gene, ID2, and RUNX2 in T-ALL cell line LOUCY. These T-ALL oncogenes are physiologically expressed in hematopoietic progenitor cells and regulate the differentiation lineage of the closely related NK-cells [8,9,33]. Due to the high regulatory impact of these genes we speculate that they contribute significantly to the separate positioning of LOUCY after cluster analysis of T-ALL cell lines.

We showed that HOXA9 and HOXA10 activated ID2 in T-cells (and HELA) while RUNX2 was not regulated by these homeodomain proteins. Coexpression of HOXA9/HOXA10 and ID2 was detected in cell lines expressing MLL-fusion proteins which have been shown to activate particular HOXA genes, including HOXA9 and HOXA10 [16-18]. Thus, our results highlight ID2 as an indirect target of MLL fusion proteins. However, phosphorylation of HOXA9 and HOXA10 proteins by protein kinase C reduces their DNA-binding activity [52,53]. This modification may explain the limited correlation between endogenous expression levels of HOXA10 and ID2 as observed in JURKAT cells.

ID2 belongs to the basic helix-loop-helix family of transcription factors. Physiologically, ID2 is expressed in progenitors of NK-cells and contributes essentially to the differentiation of this lymphocytic lineage [8-10]. Together with family members TAL1 and LYL1, ID2 confers the potential to suppress the activity of bHLH proteins TCF3 and TCF12. Their activity in turn has fundamental importance for T-cell development as demonstrated by differentiation arrest upon suppression [5-7]. Furthermore, TCF3 activates pro-apoptotic BIM, enhancing sensitivity to cell death during selection processes [38]. We showed that the expression levels of BIM were reduced in HOXA9/HOXA10 and ID2 overexpressing cells, presumably mediated by ID2. Accordingly, regulation of BIM by ID2 has been also described in colorectal carcinoma [54]. Finally, the NOTCH-pathway is frequently activated via various mutations in T-ALL, and ID proteins release the negative autoregulation of the NOTCH-target HES1 [55]. Therefore, deregulation of ID2 via HOXA9/HOXA10 may be a crucial step in thymocytic transformation by perturbing T-cell differentiation and apoptosis.

HOXA genes are physiologically expressed in lymphocytic progenitor cells and become downregulated during T-cell development [14,56]. During differentiation of NK-cells HOXA10 remains active, indicating a crucial role in this related lymphocytic lineage [33,57]. HOXA5, HOXA9 and HOXA10 have been identified as oncogenic targets in T-ALL samples, supporting their dominant role in lymphocyte development [30]. Clustered homeobox genes, including HOXA genes, are regulated by competing chromatin complexes which comprise activating HMTs like MLL or SET1 proteins and repressing polycomb-complexes [19,20,39-42]. By expression profiling we identified two genes coding for components of PRC2, namely EZH2 and HOP, and demonstrated their involvement in regulation of HOXA9/HOXA10 expression in NK/T-cells.

Inhibition of EZH2 by DZNep or siRNA consistently increased expression of HOXA10 while its activation by rapamycin decreased HOXA10 levels. The latter result may influence the treatment of HOXA-positive T-ALL patients because of frequent aberrant activity of the AKT-pathway in this disease [58]. T-ALL cell line LOUCY lacks EZH2 expression and, coherently, showed no effects after rapamycin or DZNep treatments. These data suggested that LOUCY cells have no or only restricted function of PRC2 due to absence of EZH2. Accordingly, reduction of PRC2 component EED in embryonic stem cells results in loss of repressor activity as well and subsequently in increased expression of HOXA genes [40]. In mammalian cells knockdown of PRC2 components EZH2 or PHF1 led to upregulated HOXA gene expression [59]. Furthermore, in *Drosophila *mutations in each of ten tested different PRC components, including the EZH2-homologue, results in ectopic HOX-gene expression, supporting the necessity of a complete complex for its gene repression activity [60].

By examining the cause of EZH2 silencing in LOUCY we identified reduced TFDP1 expression. Increased expression of TFDP1 in LOUCY via lentiviral transduction of an expression construct restored EZH2 expression. TFDP1 is a cofactor for E2F transcription factors, acting as heterodimers, which have been shown to regulate EZH2 transcriptional expression [50]. Therefore, TFDP1 may together with T-cell expressed E2F1 physiologically contribute to EZH2 gene activity. E2F1 regulates proliferation and apoptosis during T-cell development. Reduced E2F1 activity enhances survival of both thymocytes and T-ALL cells [29,61]. Therefore, we suggest that low expression levels of TFDP1 may additionally restrict E2F1-mediated apoptosis in T-ALL cells. Interestingly, TFDP1 has also been shown to interact via E2F6 with EPC1 [62], demonstrating the lack of another component of PRC2 in this T-ALL cell line. Recently, deep sequencing analysis discovered mutated and consequently inactive EZH2, identifying EZH2 as a tumor suppressor gene in difffuse large B-cell lymphoma [63].

HOP has also been shown to interact with PRC2 component EPC1 [47]. Forced expression of HOP in JURKAT cells and reduced expression in NK-cell lines decreased and increased expression of HOXA10, respectively, demonstrating an inhibitory effect in T-cells and NK-cells. Therefore, these results indicated that HOP mediated repression of HOXA10 by modulation of PRC2 activity. Accordingly, NK-cell line NK-92 expressed higher levels of HOP and lower levels of HOXA9/HOXA10 in comparison to YT cells, supporting a repressive role of HOP. This mode of HOX gene regulation seems to be maintained during the development of NK-cells and deactivated in that of T-cells because HSCs were HOP positive. Surprisingly, HOP-overexpressing T-cells were significantly more sensitive to DZNep-treatment. This observation confirmed an effect of HOP on PRC2 and may suggest that HOP mediated conformational changes within this complex, resulting in enhanced repressive activity concomitantly facilitated by an increased accessibility of DZNep to its binding site.

**Figure 15:**
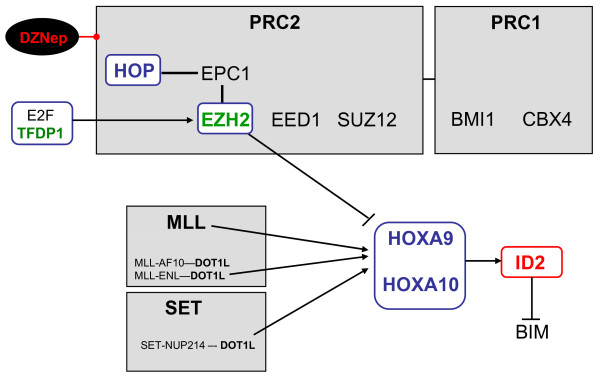
**Network of HOXA-regulation**. The diagram depicts major chromatin complexes, including PRC1, PRC2, MLL and SET with selected components or mutated fusion proteins which regulate the transcription of HOXA genes.

Activating chromatin complexes contain HMT activity mediated by closely related MLL/SET1 proteins [19,20]. Chromosomal translocations of MLL are frequent aberrations in both, AML and ALL, including T-ALL. Their most prominent oncogenic targets are HOXA genes [16-18]. Furthermore, Van Vlierberghe and colleagues identified recently a microdeletion in LOUCY, generating the fusion gene SET-NUP214 [21]. This fusion protein in addition to CALM-AF10, which has also been detected in T-ALL, interacts with HMT DOTL1, performing aberrant H3-methylation and concomitant HOXA deregulation [21,64]. Another fusion protein in T-ALL, SETBP1-NUP98, comprises the SET-interaction partner SETBP1 and, therefore, may also recruit HMT activity [65]. However, downstream effects of SETBP1-NUP98 have not yet been analyzed. Finally, several direct interactions have been described between activating MLL/SET1, SET and repressing PRC complexes, respectively, representing a large and complex network for regulation of HOXA in addition to other developmental genes [66-68]. Those regulative interactions together with results obtained in this study are summarized in Fig. 15. Taken together, certain components of gene regulating chromatin complexes, including MLL, SET, SETBP1 and EZH2, are oncogenic targets, representing a key mechanism of leukemic HOXA-deregulation in T-ALL. In this context, LOUCY cells may serve as a useful model system to investigate the function of PRC2 and its component EZH2 in T-ALL.

## Conclusions

Our data indicate that PRC2 contributes to the differentiation of NK/T-cells via regulation of HOXA9/HOXA10 gene expression. Deregulated function of PRC2 is involved in T-cell leukemogenesis, resulting in ectopic activation of NK-cell lineage-associated HOXA9/HOXA10 genes and their target ID2.

## Competing interests

The authors declare that they have no competing interests.

## Authors' contributions

SN designed the research and wrote the paper, LV and MS performed lentiviral cloning and transduction, VM supplied DZNep together with a protocol, CM and MK performed cell culture and labwork, RM performed cytogenetic analysis and HD wrote the paper. All authors read and approved the final manuscript.
